# Inhomogeneous energy transfer dynamics from iron-stress-induced protein A to photosystem I

**DOI:** 10.3389/fpls.2024.1393886

**Published:** 2024-05-16

**Authors:** Parveen Akhtar, Sanjib Jana, Petar H. Lambrev, Howe-Siang Tan

**Affiliations:** ^1^ Institute of Plant Biology, HUN-REN Biological Research Centre, Szeged, Hungary; ^2^ School of Chemistry, Chemical Engineering and Biotechnology, Nanyang Technological University, Singapore

**Keywords:** cyanobacteria, excitation energy transfer, ultrafast spectroscopy, iron-stress, photophysics, photosystem I, light-harvesting, iron stress

## Abstract

Cyanobacteria respond to iron limitation by producing the pigment-protein complex IsiA, forming rings associated with photosystem I (PSI). Initially considered a chlorophyll-storage protein, IsiA is known to act as an auxiliary light-harvesting antenna of PSI, increasing its absorption cross-section and reducing the need for iron-rich PSI core complexes. Spectroscopic studies have demonstrated efficient energy transfer from IsiA to PSI. Here we investigate the room-temperature excitation dynamics in isolated PSI–IsiA, PSI, IsiA monomer complexes and IsiA aggregates using two-dimensional electronic spectroscopy. Cross analyses of the data from these three samples allow us to resolve components of energy transfer between IsiA and PSI with lifetimes of 2—3 ps and around 20 ps. Structure-based Förster theory calculations predict a single major timescale of IsiA-PSI equilibration, that depends on multiple energy transfer routes between different IsiA subunits in the ring. Despite the experimentally observed lifetime heterogeneity, which is attributed to structural heterogeneity of the supercomplexes, IsiA is found to be a unique, highly efficient, membrane antenna complex in cyanobacteria.

## Introduction

Many cyanobacterial species, when exposed to iron limitation conditions, produce a specialized pigment-protein complex, iron stress-induced protein A (IsiA), that is known to assemble into either individual or concentric rings associating with photosystem I (PSI) (Bibby et al., 2001; Boekema et al., 2001; Kouřil et al., 2005). In isolated PSI–IsiA complexes IsiA efficiently transfers absorbed photon energy to the PSI core (Andrizhiyevskaya et al., 2004), which can extend the absorption cross-section of the photosystem and help reduce the number of iron-rich PSI core complexes in the cells. However, IsiA has also been suggested to have a photoprotective function (Ihalainen et al., 2005; Chen et al., 2017), dissipating excess energy or to act as a reservoir for chlorophyll (Chl) (Jia et al., 2021). More recent spectroscopic observations have substantiated the notion that IsiA serves as an excitation quencher via a cystein-mediated electron-transfer mechanism (Chen et al., 2021).

Recent high-resolution structures of isolated PSI–IsiA supercomplexes (Toporik et al., 2019; Akita et al., 2020; Cao et al., 2020; Nagao et al., 2023), bring insight into the potential pathways of excitation energy transfer (EET) from IsiA to PSI. The IsiA monomer resembles CP43 of PSII, presumably evolved from a common ancestor protein (Biswas et al., 2023), lacks the loop connecting helices V and VI. It coordinates 17 Chls, 13 of which in positions similar to CP43. Four carotenoids are positioned at the IsiA–IsiA interface and between IsiA and PSI, bridging IsiA subunits in the ring and facilitating interaction with PSI. The PsaF subunit in PSI was identified as key for the interaction with IsiA (Fromme et al., 2003). Lack of PsaF and PsaJ subunits leads to smaller, partial IsiA rings (Kouřil et al., 2003).

Spectroscopic studies of isolated PSI–IsiA complexes from *Synechococcus* sp. PCC 7942 and *Synechocystis* sp. PCC 6803 have shown rapid and highly efficient EET from IsiA to PSI (Melkozernov et al., 2003; Andrizhiyevskaya et al., 2004). The addition of IsiA extends the trapping time from 20–25 ps to 39–44 ps. The trapping time was also shown to be different depending on the number of rings surrounding PSI (Chauhan et al., 2011). Kinetic components of EET from the IsiA antenna to the PSI core complex with lifetimes between 30 and 90 ps have been resolved in several types of PSI–IsiA complexes at 77 K (Akita et al., 2020; Akhtar et al., 2023; Nagao et al., 2023). EET at physiological temperatures is expected to be considerably faster because of larger overlap between the broader emission and absorption spectra of Chls. From the difference of the transient absorption kinetics of PSI–IsiA and PSI, Melkozernov et al. (2003) extracted two lifetime components of 1.7 ps and >10 ps. They had suggested that the 1.7 ps component may represent an EET process from the IsiA antenna ring to the PSI core via closely located clusters of Chl a in IsiA and PSI, while the >10 ps may represent overall EET from the IsiA ring to PSI. In that work and since then, there have not been any conclusive proof or further studies regarding these EET dynamics.

In this work we used two-dimensional electronic spectroscopy (2DES) to study the room-temperature excitation dynamics of PSI–IsiA supercomplexes isolated from *Synechocystis* sp. PCC 6803 cells grown under iron limitation conditions. The advantages of 2DES lie in the simultaneous resolution of the transient absorption signal with respect to both excitation and detection wavelengths (Gelzinis et al., 2019) that helps to distinguish kinetic components associated with population transfer between different energy states (EET) from excitation decay. EET components are clearly identified by the appearance of symmetric cross-peaks associated with energetically uphill and downhill EET pathways; further, the establishment of thermal equilibrium among energy states is detected by following the correlation between excitation and detection wavelengths (Akhtar et al., 2017). To aid the interpretation of the 2DES data, we performed measurements on isolated PSI and IsiA under the same experimental conditions and calculations of EET applying Förster theory and kinetic model simulations. We will show that EET between IsiA and PSI is remarkably fast at least in some complexes, with lifetimes of 2—3 ps but also that the EET times can differ by an order of magnitude, which probably reflects structural and functional heterogeneity.

## Materials and methods

### Cyanobacterial strains and growth conditions

*Synechocystis* sp. PCC6803 cells were used in this study. The cells were grown photoautotrophically in BG-11 medium supplemented with 5 mM HEPES–NaOH (pH 8). Iron-stressed cultures were obtained by inoculating the cells in BG-11 medium lacking iron-containing compounds. The cultures were placed on a rotary shaker (100 rpm) at 30°C under continuous white light (~ 35 μmol photons m^−2^ s^−1^).

### Sample preparations

Thylakoid membranes isolated from one-week old cells were used as a starting material for all the samples (PSI, PSI–IsiA and IsiA). PSI was prepared following the protocol described by Akhtar et al. (2021a) and further purified by using an ion-exchange chromatography column (HiTrap Capto Q ImpRes, Cytiva, USA) on NaCl gradient (0–300 mM). PSI–IsiA and IsiA complexes were obtained by using the method described by Yeremenko et al. (2004) with small modifications. Briefly, thylakoid membranes were solubilized by incubating with 1% *n*-dodecyl-β-maltoside (β-DDM) on ice. The unsolubilized material was removed by centrifugation at 30,000 g for 30 min. The resulting supernatant was then filtered through 0.45 µm filters and subsequently loaded on an ion-exchange chromatography column (HiTrap Capto Q ImpRes, Cytiva, USA). The fractions containing PSI–IsiA were successfully eluted applying a 0–300 mM Mg_2_SO_4_ gradient, concentrated, and further purified using a size-exclusion chromatography column (HiPrep 16/60 Sephacryl S-300 HR, Cytiva, USA).

### Laser spectroscopy setup

Fourier-transform femtosecond 2DES was performed using a partially collinear “pump-probe” geometry setup (Zhang et al., 2012). The fundamental laser beam centered at 800 nm with 1 kHz repetition rate and 50-fs pulse duration was obtained from a commercial amplified Ti:sapphire laser system (Legend, Coherent). The laser pulses were focused into a 1-meter pressurized Argon tube using a 1-meter concave mirror to generate a supercontinuum. The output supercontinuum pulse was then split using a wedged glass window. The transmitted pulse which is the major fraction was used as the pump pulse and the reflected was used as the probe and reference. The pump pulse was further compressed using a single-prism compressor (Akturk et al., 2006) and passed through an acousto-optic programmable dispersive filter (AOPDF) pulse shaper unit (Dazzler, Fastlite) to generate a double-pulse train. The pulse shaper was programmed to create pulse pairs with controlled inter-pulse delay time and phase difference. The pump pulses compression and shaping, has spectral bandwidth of ~100 nm and time duration of ~17 fs, measured using an autocorrelator (PulseCheck, APE).

The probe pulse was compressed using a pair of dispersion-compensating mirrors (DCM10, Laser Quantum) and directed through a motorized linear stage (Physik Instrumente) to control the delay against the pump pulses waiting time (*T*_w_). The probe beam was further spilt (50/50), using a beam splitter, to use as a reference pulse to compensate for power fluctuations during the measurements. The polarization of the probe pulse was set to 54.7° (magic angle) with respect to the polarization of the excitation pulses using a half-wave plate and a polarizer. Finally, the pump, probe and reference pulses were spatially overlapped onto the sample unit using an off-axis parabolic mirror. The probe beam was then frequency resolved and detected using a spectrometer (Acton SP230, Princeton Instruments) assembled with a CCD camera (PIXIS 100, Princeton Instruments). The time delay τ between the two excitation pulses (coherence time) was scanned between 0–150 fs with a 3-fs step. The waiting time (*T_w_*) between the excitation and probe pulses was scanned from −100 fs to 600 ps in a quasilogarithmic progression.

The 2D photon echo signals were recorded by utilizing a 2-phase cycling scheme (φ = 0, 180°) in a partially rotating frame of reference (Zhang et al., 2012). The signals (2D interferograms) were Fourier-transformed along τ to obtain 2D electronic spectra in the frequency/wavelength domain (with excitation/detection wavelengths λ_τ_/λ*_t_*).

## Results

### Excitation dynamics of IsiA complexes

The excitation dynamics of isolated IsiA complexes, PSI complexes and PSI–IsiA supercomplexes was measured by recording series of room-temperature 2D electronic spectra at different waiting times (Tw) in a 600 ps window. For a direct comparison, measurements were done on complexes isolated from the same cell cultures and under the same measurement conditions, using spectrally broad excitation pulses that completely cover the Chl Q_y_ absorption bands of all sample types (**Supplementary Figure 1**). The sample integrity was monitored by recording absorption spectra before and after the 2DES measurements (**Supplementary Figure 2**). We begin with analysis of IsiA complexes, which were studied to establish the dynamic spectral features of IsiA in the absence of coupling to PSI.

IsiA can be isolated in aggregated or monomeric solubilized state and its spectroscopic properties can vary depending on the sample preparation and conditions (Biswas et al., 2023). The monomeric IsiA complexes had absorption spectra with a maximum at 670 nm, whereas IsiA aggregates displayed an absorption maximum at 672 nm and a shoulder around 682 nm (**Supplementary Figure S1**). To test which state represents more closely the spectral features of IsiA in the PSI–IsiA supercomplex, we compared the absorption spectra of PSI–IsiA and IsiA aggregates and monomers. The weighted difference of the absorption spectrum of PSI–IsiA minus PSI, scaled assuming a PSI_3_IsiA_18_ stoichiometry, resembled closely the absorption spectrum of IsiA monomers except that the difference spectrum was redshifted by about 1 nm (**Supplementary Figure 3**). It could be said that, in the supercomplex, IsiA adopts features of both the monomeric and aggregated state. In the following, we present 2DES experiments on both monomeric and aggregated IsiA.

**Figure 1:**
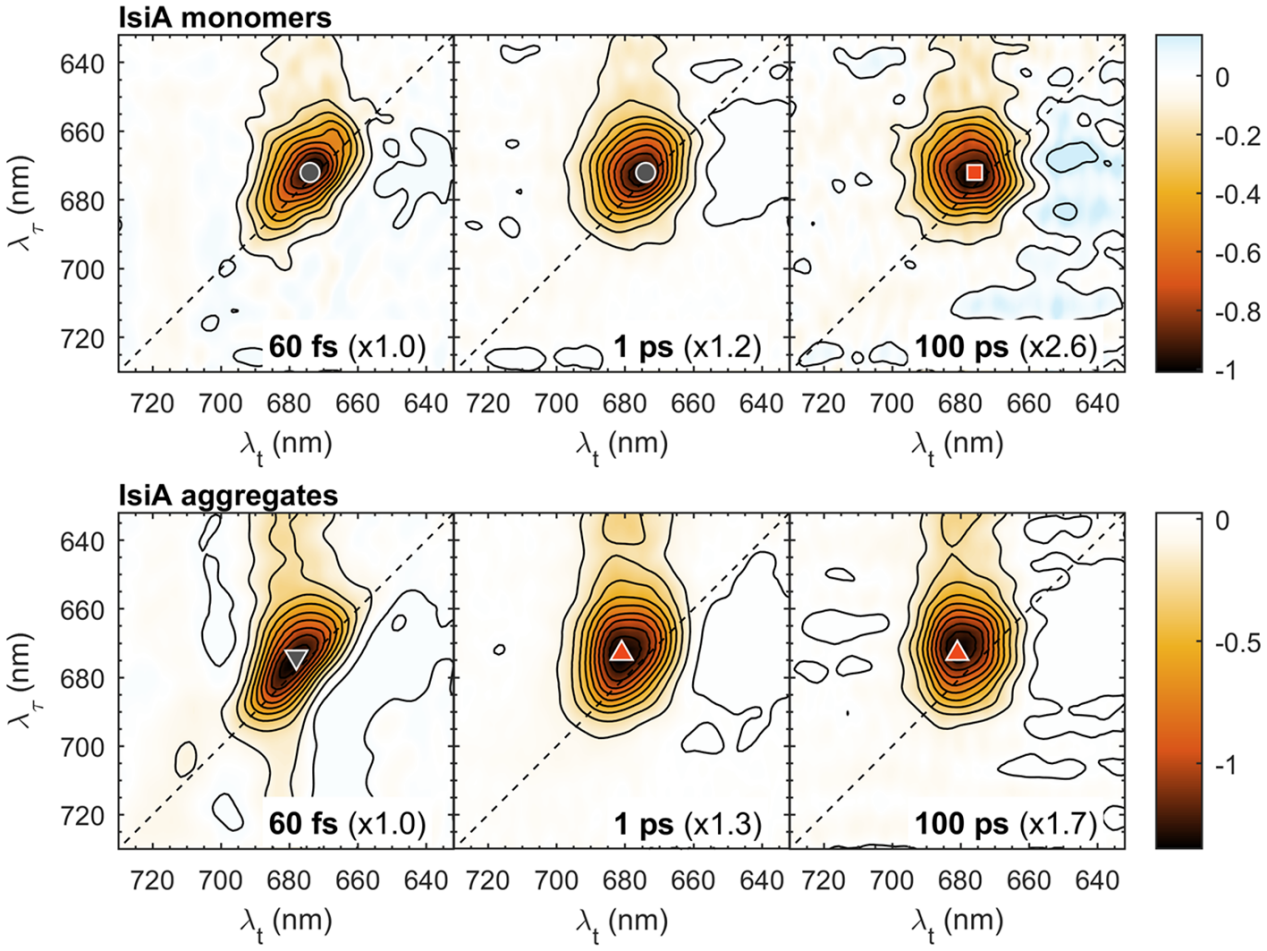
Selected 2D electronic spectra of isolated IsiA monomers and aggregates at waiting times *T_w_* = 60 fs, 1 ps and 100 ps. The vertical axis represents excitation wavelength (λ_τ_) and the horizontal axis represents the detection wavelength (λ*_t_*). The colour scale represents the purely absorptive 2D signal with contours marking 10% increments. For easier comparison, the spectra are multiplied by scaling factors, indicated in parentheses. The coloured symbols mark selected λ_τ_→λ*_t_* peak positions: 672→674 nm (grey circle), 672→676 nm (red square); 673→678 nm (grey downward triangle), 673→681 nm (red upward triangle).

**Figure 1** shows 2D electronic spectra of IsiA monomers and aggregates at waiting times *T_w_* = 60 fs, 1 ps, and 100 ps. Note that in all 2D spectra the vertical axis represents the excitation wavelength (λ_τ_) and the horizontal axis represents the detection wavelength (λ*_t_*). In this representation, horizontal slices of the 2D spectra are equivalent to conventional transient absorption spectra.

At the earliest waiting time (*T_w_* = 60 fs), the spectra are predominantly diagonal because of self-correlation of the excitation and detection wavelengths in the absence of EET. Weak off-diagonal signals observed at λ_τ_ < 660 nm reflect predominantly ultrafast vibronic and Q_x_–Q_y_ relaxation. Low-amplitude signals at λ_τ_ > 700 nm in the 60 fs of IsiA aggregates are remaining pulse overlap artefacts. At longer *T_w_* the spectrum becomes markedly broader along the detection wavelength λ*_t_* as excitations are spread over different energy levels in IsiA. In monomeric IsiA, the maximum bleaching signal shifts only slightly to longer λ*_t_* – from 674 nm at *T_w_ =* 60 fs (marked with a grey circle) to 676 nm at *T_w_* = 100 ps (red square). In aggregates, a more pronounced redshift to 681 nm is observed due to the population of the long-wavelength Chl states mentioned above. However, considerable signal intensity persists at wavelengths shorter than 680 nm in both IsiA monomers and aggregates.

**Figure 2:**
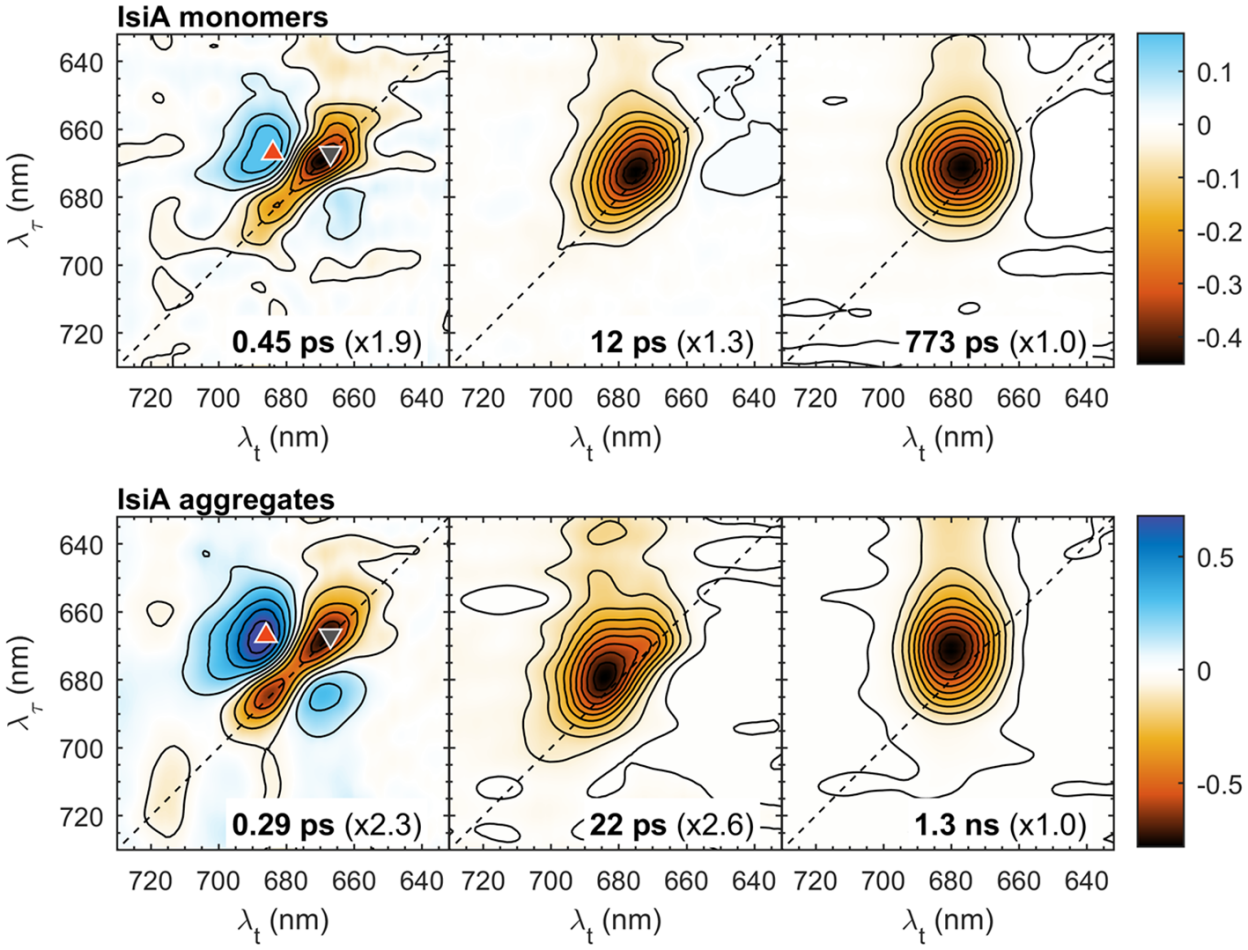
2D decay-associated spectra (2D DAS) of isolated IsiA monomers and aggregates resulting from global analysis of the 2DES data in the time range from 60 fs to 600 ps with three exponential decay lifetimes, indicated in the panels. The colour scale represents the pre-exponential amplitudes. For easier comparison, the spectra are multiplied by scaling factors, indicated in parentheses. The coloured symbols mark selected λ_τ_→λ*_t_* peak positions: 667→667 nm (grey downward triangle), 667→686 nm (red upward triangle).

To get more information about the kinetics of spectral evolution in IsiA, global multiexponential lifetime analysis was performed on the traces at all excitation/detection wavelengths. Three decay components were sufficient to fit the kinetics in the range from 60 fs to 600 ps The goodness of fit was evaluated by comparing the resultant χ^2^ statistics and inspecting the fit residuals at different wavelengths (**Supplementary Figure 4**) to confirm the lack of trends above the noise floor. The decay lifetimes were 0.45 ps, 12 ps and 0.8 ns for IsiA monomers and 0.3 ps, 22 ps and 1.3 ns for aggregates. The corresponding 2D decay-associated spectra (2D DAS) representing the pre-exponential amplitudes at different λ_τ_ and λ*_t_* for each decay component are plotted in **Figure 2**. In either sample, the three spectra reveal markedly different features of the corresponding kinetic components. The shortest-lived component is characterized by negative peaks along the diagonal line flanked by positive off-diagonal peaks, which indicates excitation transfer between different energy levels. The negative peaks (e.g. at 667 nm, marked by a downward triangle) represent the decay of initially excited states and the positive peaks (667→686 nm, upward triangle) show the rising population of acceptor states. Note that at room temperature EET is bidirectional resulting in positively-signed peaks on both sides of the diagonal line (Akhtar et al., 2017). Thus, the 0.3–0.4 ps component is associated with the main timescale of energy equilibration within the IsiA complexes.

The second and the third 2D DAS, with lifetimes of 12/22 ps and 0.8/1.3 ns, respectively, have all-negative amplitudes; therefore, they can be attributed to the overall decay of excitations in IsiA that occurs with two characteristic timescales. The multi-exponential excitation decay of IsiA is well known and is attributed to constitutive nonphotochemical quenching (Chen et al., 2017, 2021). From the relative amplitudes (scaling factors in **Figure 2**), it can be seen that a larger fraction of excitations decay with the intermediate lifetime in monomers than in aggregates. A more accurate quantitative description of the decay kinetics can be obtained by time-resolved fluorescence (Akhtar et al., 2023). Here we note that the shape of the 12/22 ps 2D DAS, more specifically its dependence on the excitation wavelength λ_τ_, indicates that spectral equilibration is not yet complete and is still undergoing on this timescale in both monomers and aggregates. In contrast, the final 2D DAS shows virtually no excitation dependency of the signal. In monomers, the spectral shape approximates a 2D gaussian centred at λ*_t_* = 676 nm. For aggregates, the spectrum is broader along λ*_τ_*, reflecting the wider range of exciton energy levels, and is centred at λ*_t_* = 681 nm.

### 2D electronic spectra of PSI-IsiA and PSI

**Figure 3:**
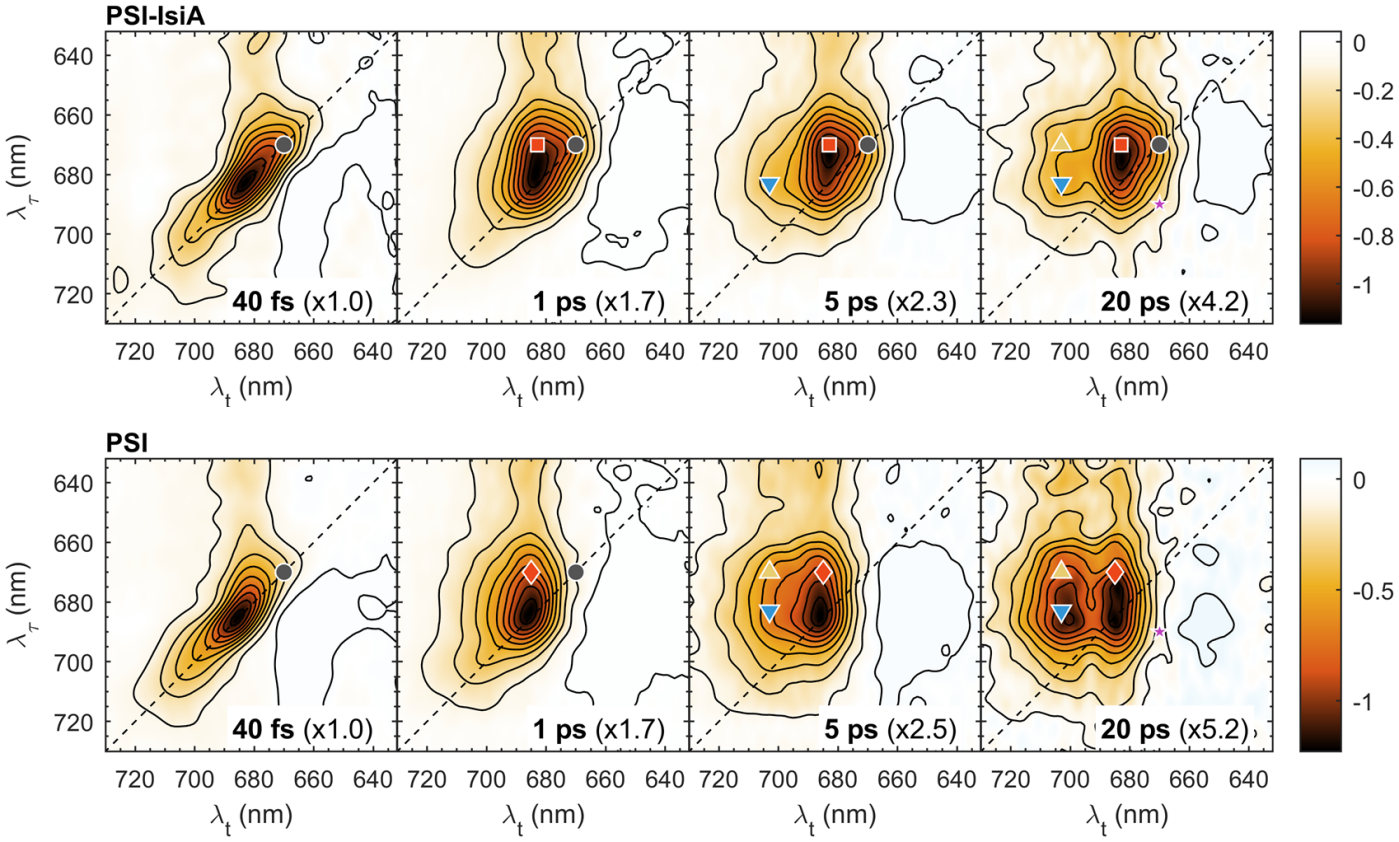
Selected 2D electronic spectra of PSI–IsiA and PSI complexes recorded at room temperature and waiting times *T_w_* = 0.04, 1, 5, and 20 ps. The vertical axis represents excitation wavelength (λ_τ_) and the horizontal axis represents the detection wavelength (λ*_t_*). The colour scale represents the purely absorptive 2D signal with contours marking 10% increments. For easier comparison, the spectra are multiplied by scaling factors, indicated in parentheses. The colored symbols mark selected λ_τ_→λ*_t_* peak positions: 670→670 nm (grey circle), 670→683 nm (red square), 670→685 nm (red diamond), 670→703 nm (yellow upward triangle), 683→703 nm (blue downward triangle), 690→670 nm (purple star).

Next, we compare the 2D electronic spectra of PSI–IsiA and PSI complexes, which are plotted side-by-side in **Figure 3**. The spectra at *T_w_* = 40 fs are predominantly diagonal with a vibronic relaxation cross-peak at λ_τ_ ≤ 660 nm. At longer *T_w_* the diagonal signal intensity drops giving way to off-diagonal signals (cross-peaks) as excitations shift to different Chls. In PSI–IsiA, the appearance of a cross peak at λ_τ_ ≈ 670 nm and λ*_t_* ≈ 683 nm (670→683 nm, marked with a red square symbol in **Figure 3**) is evident at *T_w_* = 1 ps. A corresponding 670→685 nm cross-peak appears in PSI (marked with a red diamond) and the diagonal peak at 670 nm (grey circle) disappears as excitations are transferred to a pool of Chls absorbing around 685 nm. In contrast, the diagonal signal remains prominent at longer *T_w_* in both PSI–IsiA and IsiA (c.f. **Figures 1**, **3**), and therefore can be used as a marker for the excited population of IsiA. On the other hand, the bleaching signal is strongly diminished at 5 and 20 ps in PSI–IsiA, whereas it is long-lived in IsiA (cf. **Figure 2**). Therefore, the time-dependent decay of the diagonal signal at 670 nm can be taken as one indicator of EET from IsiA to PSI.

The 2D spectra of PSI trimers show that thermal equilibration in the wavelength region 660–700 nm is virtually completed by *T_w_* = 5 ps, since the spectral shape is independent of the excitation wavelength. In contrast, the 2D spectra of PSI–IsiA show a clear dependence on λ_τ_ up to 20 ps – note the remaining diagonal peak at 670 nm, indicating remaining excited IsiA, and the cross-peak at 683→703 nm (blue triangle in **Figure 3**), showing that long-wavelength states are preferentially populated by EET from the core PSI antenna rather than from IsiA.

**Figure 4:**
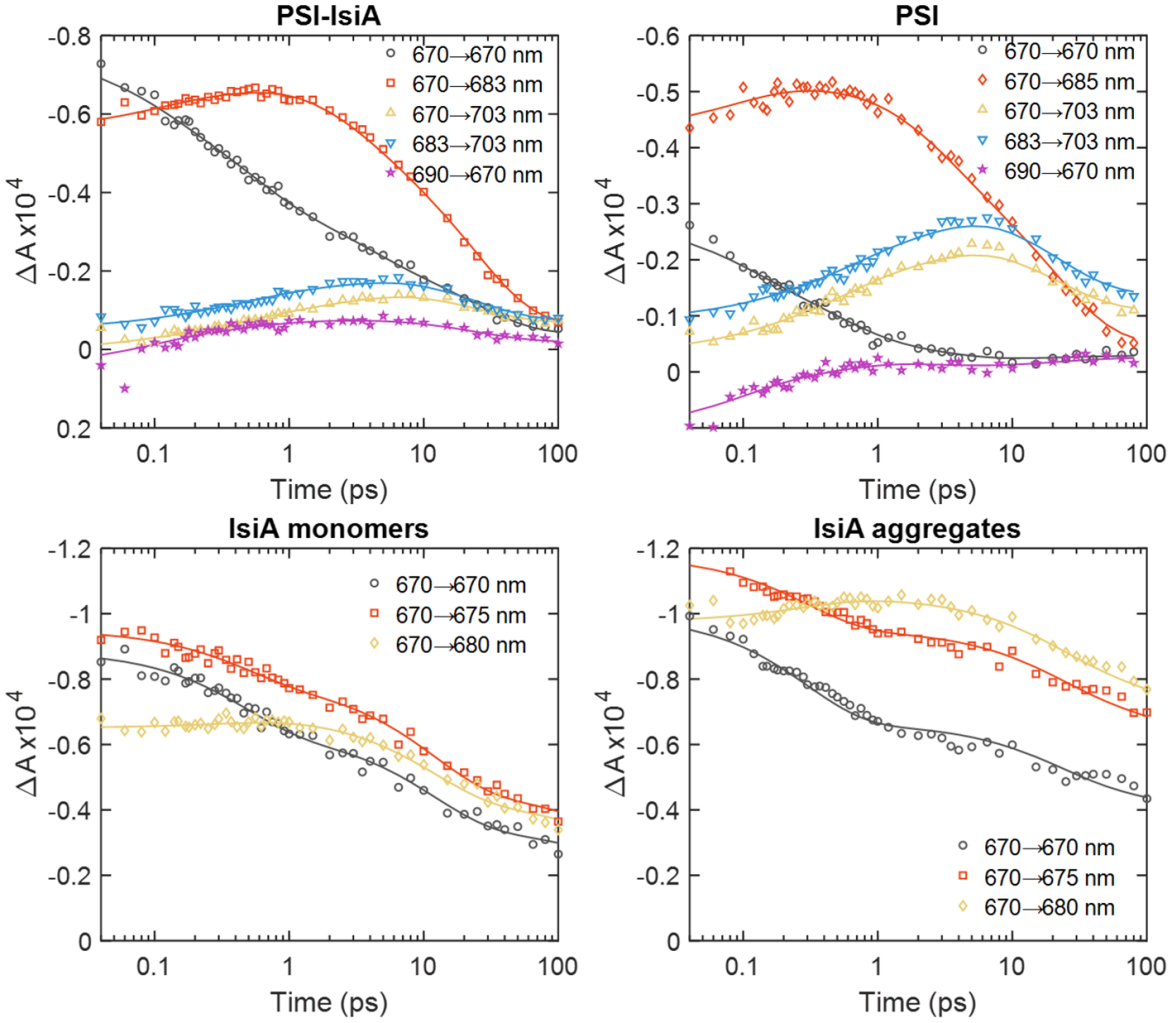
Waiting-time dependence of the 2D absorptive signal measured from PSI–IsiA, PSI, and monomeric IsiA complexes at selected excitation/detection wavelengths λ_τ_→λ*_t_*. The experimental data points are indicated with the symbols and the lines are obtained by global lifetime analysis of the kinetics. Note the logarithmic time scale.

**Figure 4** compares the time-dependent traces of the 2D signal at the diagonal and off-diagonal points marked in **Figure 3**. It can be seen that the signal at 670 nm in PSI–IsiA (grey circles) decays over different timescales from 0.1 to 100 ps. In contrast, PSI exhibits little bleaching at 670 nm that becomes negligible by 5 ps. This shows that IsiA population in the supercomplex has dynamics spanning the time window of up to tens of ps but is entirely depleted by 100 ps. It is also notable that the 670→703 nm trace (yellow triangles) in PSI–IsiA decay more slowly than the trace at 683→703 nm (blue triangles) at *T_w_* > 10 ps. This is an indication of slow transfer of excitations from IsiA to the long-wavelength Chls in PSI. No such excitation wavelength dependence is observed in PSI. In isolated IsiA, the bleaching signal decays considerably lesser in the same time period than in PSI–IsiA at all wavelengths, affirming that the decay of IsiA population in the supercomplex can be assigned to energy transfer rather than the intrinsic quenching present in IsiA. Lastly, the 690→670 nm trace in PSI–IsiA (purple stars in **Figure 4**) shows small but discernible rise and subsequent decay of bleaching, suggesting that excitations are transferred uphill from the PSI core to IsiA before being trapped again. In the PSI core complexes, this trace shows only ultrafast decay of positively signed excited-state absorption.

**Figure 5:**
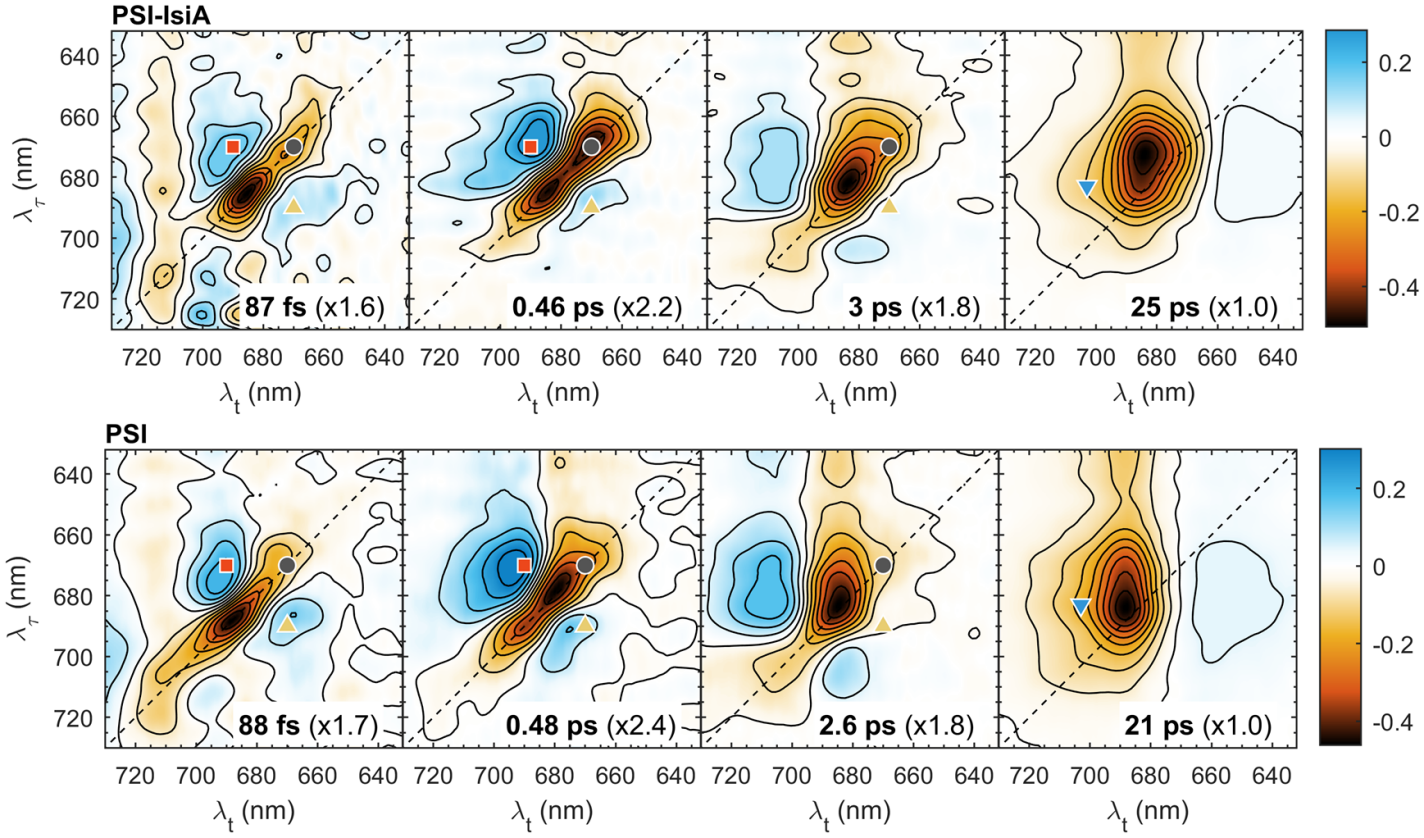
2D decay-associated spectra obtained by global analysis of the 2DES data of PSI–IsiA and PSI complexes. The lifetimes are indicated on the plots. The spectra are normalized by the scaling factors in parenthesis. Red/blue colours indicate the decay/rise of the negative bleaching signal. The non-decaying component representing the final charge-separated state (P_700_^+^) is not shown.

Global analysis of the kinetics of PSI–IsiA and PSI resolved four decay lifetime components plus a non-decaying component for both sample types. The 2D DAS, representing the amplitudes of the decay components, are shown in **Figure 5** and the spectrum of the non-decaying component in **Supplementary Figure 5**. The decay lifetimes of PSI–IsiA and PSI complexes were very similar – 90 fs, 0.4–0.5 ps, 2–3 ps and 20–25 ps. The first three 2D DAS have features of spectral diffusion or energy transfer – negative amplitudes (marked in red), generally along the diagonal, flanked by positive-amplitude wings (in blue) at off-diagonal regions. The shortest-lived component (90 fs) probably represents fast relaxation among strongly coupled Chl exciton states, together with spectral diffusion due to fluctuations in the exciton energies and some spurious artefacts outside of the Chl absorption bands likely due to pulse overlap at early *T_w_*. The 0.4–0.5 ps component in PSI mainly represents exciton relaxation in the bulk antenna – the disappearance of the diagonal signal around 670 nm and the formation of the cross-peak around 670→690 nm. The symmetrically located cross-peak around 690→670 nm shows the corresponding uphill transfer pathways. In PSI–IsiA the diagonal peak is at 670 nm and the main cross-peak at 670→688 nm, showing that EET in IsiA also contributes to this 2D DAS. This is corroborated by the global analysis of IsiA aggregates, showing the rise of a cross-peak at 667→686 nm with a lifetime of 0.3 ps (upward triangle in **Figure 1**). From this we construe that the 0.5 ps lifetime represents mainly EET processes within PSI and IsiA rather than between them.

The next decay component with a lifetime of 2.5–3 ps is dominated by equilibration of the bulk antenna Chls and the long-wavelength forms (Melkozernov et al., 2000; Savikhin et al., 2000; Gobets and Van Grondelle, 2001; Akhtar et al., 2021b). In PSI, the 2D DAS show very little contribution from diagonal signals around 670–675 nm. In PSI–IsiA, however, the 3-ps 2D DAS also features decay of diagonal signals around 670 nm, clearly showing that IsiA population decays on this timescale. No similar dynamic component is present in IsiA alone (**Figure 2**), hence the decay can be interpreted as EET from IsiA to PSI. The assignment is corroborated by the weak but nonetheless visible positively valued cross-peak at 695→670 nm showing uphill EET (presumably from PSI to IsiA).

The final decay component with a lifetime of 21 ps in PSI and 25 ps in PSI–IsiA shows mainly trapping of excitations by the PSI reaction centre. In PSI this component shows no dependence on λ_τ_ and no features of EET (the weak positive signals at λ*_t_* around 640–660 nm indicate the decay of induced absorption). This is not the case with PSI–IsiA where the 2D DAS is clearly dependent on λ_τ_ showing that the remaining population of IsiA is not completely equilibrated with PSI. Superficially, one might construe that the ~20 ps 2D DAS of PSI and IsiA (**Figure 2**) add together to give rise to the 25 ps 2D DAS of PSI–IsiA, hence, the spectral features could be explained without assuming IsiA—PSI energy transfer on this timescale. If this would be the case, however, there should be a longer-lived component representing the decay of IsiA excitations. Not only is such longer-lived decay absent from the data but IsiA population is virtually non-existent in the non-decaying 2D DAS of PSI–IsiA supercomplexes (see below). The absence of long-lived IsiA decay components affirms that excitations are transferred to and ultimately trapped by PSI, consistent with time-resolved fluorescence spectroscopy data (Akhtar et al., 2023).

**Figure 6:**
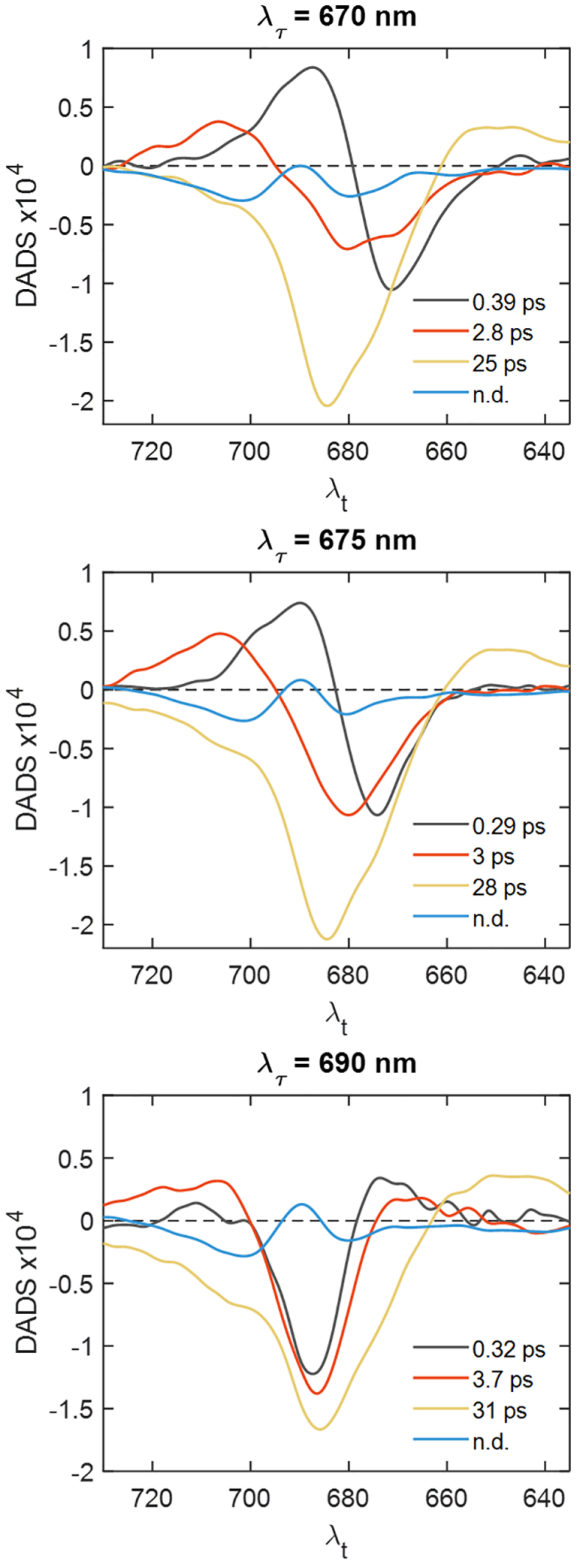
Decay-associated spectra obtained by global analysis of horizontal slices of the 2D electronic spectra of PSI–IsiA at different excitation wavelengths λ_τ_.

As an alternative approach to identify the timescales of the EET processes in PSI–IsiA, we extracted horizontal slices of the 2D electronic spectra at different excitation wavelengths – predominantly exciting IsiA or PSI (**Supplementary Figure 6**) – and analyzed them separately. The resulting decay-associated spectra are plotted in **Figure 6**. The analysis for λ_τ_ = 670 nm and λ_τ_ = 675 shows a 3-ps component with sizeable negative DAS amplitudes at λ*_t_* < 680 nm, whereas the corresponding DAS of PSI has a negative maximum at 682 nm (**Supplementary Figure 7**), confirming that excited IsiA population decays with a 3-ps lifetime in PSI–IsiA. It is also of note that the relative amplitudes of the 3-ps DAS at λ*_t_* in the range 670—675 nm depend on λ_τ_, indicating that energetic equilibration within IsiA is not complete on this timescale. A strong indication that PSI–IsiA EET occurs on a 3-ps timescale is that the DAS for λ_τ_ = 690 nm (**Figure 6**) has a negatively signed peak at λ*_t_* ≈ 690 nm and a positively-signed peak at λ*_t_* ≈ 670 nm – showing uphill excitation transfer to IsiA.

The 25-ps DAS in PSI–IsiA has a pronounced shoulder around 675 nm signifying that a fraction of IsiA remains excited on the timescale of photochemical trapping. Further, the relative amplitude of the IsiA bleaching band varies with excitation wavelength λ_τ_, confirming that the equilibration between IsiA and PSI is not complete. We also find that the trapping lifetime depends on λ_τ_ too, suggesting the co-occurrence of multiple processes with similar lifetimes. Yet another observation pointing to the same conclusion is that the DAS amplitude around 710 nm depends on λ_τ_, a higher population of red Chls is observed when the excitation light is predominantly absorbed by PSI rather than IsiA.

The DAS in **Figure 6** also show the spectrum of the non-decaying component, which has negative maxima at ~680 and 700 nm and a positive maximum around 690 nm closely resembling the absorption difference spectrum of PSI with oxidized minus reduced reaction centre (Savikhin et al., 2000). It can be ascribed primarily to the transient absorbance of the oxidized Chl cation P_700_^+^. It is evident that this component is virtually independent of excitation wavelength (see also **Supplementary Figure 5**) and contains no significant contribution from IsiA, confirming that all IsiA complexes are energetically connected to a functional PSI core.

## Discussion

### Spectral evolution dynamics in IsiA monomers and aggregates

The spectral distinction between IsiA and the PSI core complex is the basis to follow the migration of excitation energy between them in the PSI–IsiA from *Synechocystis* sp. PCC 6803, studied here. While it is well established that IsiA absorbs light at shorter wavelengths than PSI, there is substantial spectral overlap between them that, combined with the excitation dynamics within the respective antenna systems, makes quantitative treatment of the dynamics of energy transfer non-trivial. Another complication is the spectral variability of isolated IsiA, as reviewed by Biswas et al. (2023). For instance, the existence of red-shifted Chl states in IsiA aggregates but not in monomers, which is apparent from their absorption spectra, has been shown by fluorescence spectroscopy of IsiA aggregates at 5 K (Van Der Weij-De et al., 2007) and, more recently, by 5 K absorption and hole-burning spectroscopy of IsiA monomers, aggregates and PSI–IsiA (Reinot et al., 2022). It is not self-evident which of these spectroscopic features reflect the intact PSI–IsiA supercomplex. It is tempting to assume that IsiA aggregates more closely represent the state of IsiA in the supercomplex, which is itself oligomeric. The results of Reinot et al. (2022) support the existence of a low-energy state in PSI–IsiA, absorbing at 683 nm, which is presumably missing in monomers due to the loss of Chls. Further, they propose that the low-energy Chls are located at the interface between IsiA and the PSI antenna facilitating energy transfer between them. However, we could better fit the experimental absorption spectra of PSI–IsiA with a sum of PSI and monomeric IsiA, which lacks the red-shifted absorption around 683 nm. This seeming discrepancy may be a result of different sample preparations.

Our 2DES experiments, showed that spectral equilibration occurs on comparable timescale of 0.3—0.45 ps within both IsiA monomers and aggregates. After initial equilibration, the excitations are spread over Chls absorbing at a broad range of wavelengths between 670 and 690 nm with lasting bleaching signal on the short-wavelength side in both aggregates and monomers. Since no bleaching signal is observed at 670 nm in PSI after about 1 ps, the bleaching at 670 nm is used to monitor the population of IsiA in the supercomplex.

### Two timescales of energy transfer from IsiA to PSI

The 2DES data presented here shows evidence that IsiA effectively transfers energy to the PSI core in PSI–IsiA complexes. With the help of global lifetime analysis, we could resolve a lifetime of 2.5—3.7 ps that shows, depending on the initial excitation, either downhill and uphill components of energy equilibration between Chl pools absorbing around 670 and 690 nm. As this component is neither present in isolated PSI nor in IsiA, it must be assigned to the migration of excitations between IsiA and PSI. The results build upon the transient absorption spectroscopy study of Melkozernov et al. (2003) who first reported the decay of IsiA bleaching with a 3 ps lifetime in PSI–IsiA after excitation at 665 nm and tentatively assigned this kinetic component to coupling between IsiA and the PSI core Chls. Here we reinforce their assignment by revealing the spectral equilibration dynamics of isolated IsiA and by detecting the back transfer from PSI to IsiA thanks to the 2DES technique.

The extremely rapid EET from such a large antenna system containing more than 200 Chls is notable. It exceeds by orders of magnitude the timescale of EET from the phycobilisomes to PSI, which has recently been determined to be in the range of 200 ps in *Synechocystis* sp. PCC 6803 (Biswas et al., 2024). It is also significantly faster compared to EET from the peripheral light-harvesting antenna complex of PSI in higher plants (Akhtar et al., 2018; Croce and Van Amerongen, 2020).

Apart from the well resolved ~3 ps EET, the 2DES spectra indicate that a population of IsiA transfers energy on a slower timescale, with a lifetime in the range of 25 ps. We infer the existence of slower equilibration by the inclined shape of the corresponding 2D DAS showing a pronounced correlation between λ_τ_ and λ*_t_*. It is worth noting that the 25 ps 2D DAS probably represents several co-occurring processes with similar lifetimes that cannot be explicitly resolved. The main contribution to this lifetime is evidently the overall excitation trapping by the PSI reaction centre. It must, however, be said that a more accurate measurement of the trapping lifetime by time-correlated single photon counting gives a value of 42 ps (Akhtar et al., 2023). There are several probable causes for the difference in the trapping lifetime, apart from merely an error of measurement. One is that the 2DES measurements are affected by singlet-singlet annihilation in complexes that have acquired multiple excitations during the same pulse. At the present experimental conditions using 1 nJ per excitation pulse, annihilation is unavoidable as we estimate between 1 and 2 excitations per PSI–IsiA complex (based on the maximal transient absorption signal), i.e. 25—30% probability for creating more than one excitation in the same complex. Equilibration between IsiA and PSI could also be contributing to a shorter apparent trapping lifetime in 2DES. To this extent it is worth noting that analysis of the data at excitation wavelengths preferentially absorbed by PSI yields a longer trapping lifetimes (>30 ps). This leads us to believe that the lifetimes observed with 670 nm excitation, which predominantly excites IsiA, are shorter because of an equilibration component, in the range of 10—20 ps, that cannot be resolved separately from the main trapping.

### Structure-based interpretation of the energy transfer dynamics

To aid the interpretation of the experimental results, we turn to the molecular structure of PSI–IsiA. The cryoEM structure of the PSI_3_-IsiA_18_ complex isolated from *Synechocystis* sp. PCC 6803 has recently been reported by Harris et al. (Harris et al., 2023). The authors used the structure to perform simplified calculations of the Förster EET rate between Chl pairs, using a generic value for the spectral overlap of the donor emission and acceptor absorption spectra and neglecting any exciton interactions or effects of the local environment on the electronic couplings or pigment spectral properties. While the simplified theory cannot faithfully reproduce the supercomplex kinetics, nor give an accurate prediction of the EET rates, it is a useful tool to qualitatively understand the system dynamics and the major pathways of EET. As an indicator of the overall PSI–IsiA EET, the authors determined the sum of all microscopic rate constants between IsiA and PSI to be 2 ps^–1^. It must be stressed, however, that this number is not equivalent to any experimental observable. Rather, the observable population transfer times generally depend on all microscopic rate constants in the coupled system.

**Figure 7:**
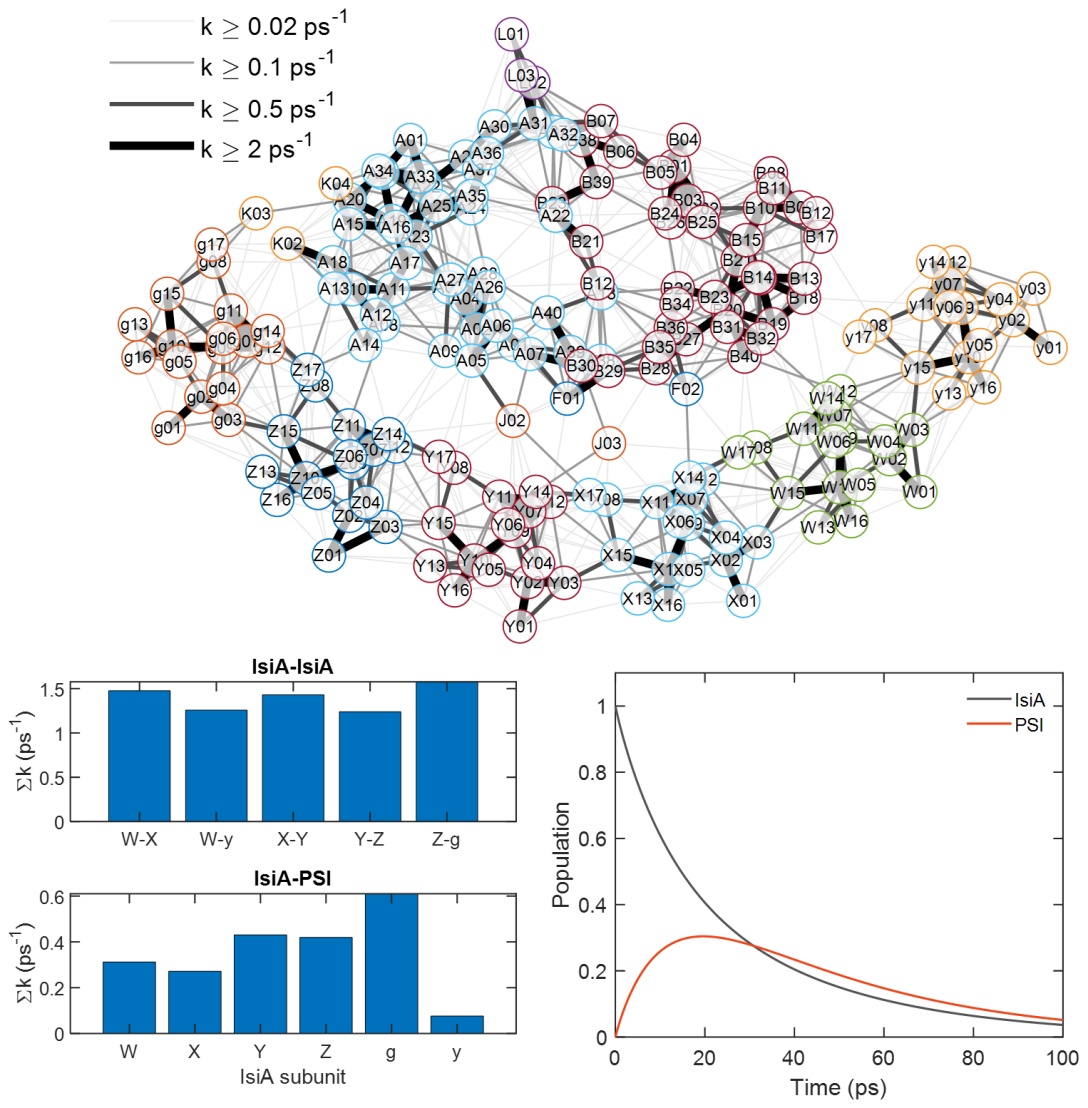
Förster energy transfer calculation of PSI–IsiA. Top: Connectivity map of the PSI_1_-IsiA_6_ complex based on the cryoEM structure (Harris et al., 2023). Alphanumeric codes denote Chls (PDB 7UMH) with letters A—L indicating subunits of PSI and W,X,Y,Z,g,y – IsiA. The lines connect Chl pairs where the calculated Förster rate constant is greater than 0.02 ps^–1^. Thicker lines indicate faster Förster EET. Bottom left – summed rate constants between different IsiA subunits and from IsiA to PSI. Bottom right – calculated time dependence of the combined population of all IsiA subunits and all PSI subunits after uniform excitation of IsiA. See **Supplementary Information** for details.

For a better understanding of the calculation result in terms of population transfer times, we extended it with calculation of the population dynamics of the system (see **Supplementary Information** for details). As a first step, we recalculated the Förster rates using the same methodology as in Harris et al (Harris et al., 2023). For simplicity, we consider only one PSI protomer with the six corresponding IsiA subunits (PSI_1_-IsiA_6_). The result is summarized in **Figure 7** as a connectivity map, where faster EET between Chl pairs is indicated by thicker lines. It is immediately apparent from the map that Chls within the PSI core and within the IsiA ring are strongly interconnected whereas the energetic connections between IsiA and PSI are relatively weaker. The summed Förster rates between IsiA subunits and between IsiA and PSI are broken down by subunit in the bar plots (**Figure 7**). The total summed EET rate from IsiA to PSI is 2 ps^–1^ as reported by (Harris et al., 2023) and the total summed rate for an individual IsiA subunit to the core is 0.3—0.6 ps^–1^ except for the weakly connected IsiA subunit y. On the other hand, the total EET rate from any IsiA subunit to neighboring IsiA is 2—3 ps^–1^.

It is interesting to note that Chls 8, 14, and 17 on IsiA that are most strongly involved in IsiA-PSI transfer also connect IsiA subunits (**Supplementary Table S1**). Chls 8 and 17 (which incidentally are only found in IsiA but not in the homologous CP43 complex of PSII), are proposed as degenerate lowest-energy exciton states in IsiA (Toporik et al., 2019; Reinot et al., 2022). These Chls could be thought of as key mediators of EET.

From the above analysis, we can make the following predictions. First, EET equilibration within the IsiA ring is considerably faster than EET from IsiA to PSI and has little influence on the effective migration time to PSI. Second, all IsiA subunits, except y, contribute comparably to the effective migration time. To test these predictions and determine the effective EET migration time, we solve the rate equation model with initial excitations placed on IsiA only. The resulting combined population of IsiA (all subunits) and PSI is plotted in **Figure 7**. The kinetics are remarkably simple, dominated by only two lifetimes – 12 ps and 37 ps (the amplitudes of the other eigenvalues of the rate matrix nearly cancel out when summed over subunits). The 12 ps lifetime represents the excitation equilibration between IsiA and PSI. On this timescale IsiA population decays and PSI population rises. The 37 ps lifetime is the overall trapping of the equilibrated excitations by the reaction centre. Although the exact lifetimes of the actual system cannot be reliably estimated by such a simplified EET model, the qualitative result is informative.

The model shows that PSI–IsiA EET is not rate-limited by excitation equilibration within the IsiA ring. Artificially increasing all EET rate constants within and between IsiA by a factor of 10 has a negligible effect on the IsiA–PSI excitation migration time (**Supplementary Table S2**). Another hypothesis, considered by Harris et al. (2023) and Reinot et al. (2022) is that EET between IsiA and PSI primarily occurs through a few bridging Chl pairs. In agreement with the two cited works, our calculation identifies that the most strongly coupled PSI—IsiA Chl pairs are g17—K03, g08—K03, Z17—A14, Y14—J03, and X14—F02 (**Figure 7**, **Supplementary Table S1**). However, setting the EET rates to zero for all those Chl pairs results in a marginal increase of the overall EET migration time to 13 ps. Another hypothesis is that EET is primarily routed through a single IsiA subunit (Reinot et al., 2022), e.g. between g and K. This hypothesis is also at odds with the model calculation. Disabling all routes out of the g subunit only increases the equilibration time to 16 ps, and any other IsiA subunits have an even smaller influence. Thus, it can be said that multiple Chls in multiple IsiA subunits contribute together to the rapid IsiA–PSI equilibration. This redundancy is probably the main reason for the “structural robustness” of EET (Harris et al., 2023), and could be regarded as a general design feature of photosynthetic units.

Note that in all these calculations, a decay rate constant of (100 ps)^–1^ was set for IsiA, to simulate the non-photochemical quenching typically observed in isolated IsiA (Chen et al., 2017; Akhtar et al., 2023). Recently, a mechanism of quenching has been proposed that involves Chls in the vicinity of Cys260 (Chen et al., 2021). Setting the quencher site to a single Chl in IsiA – e.g. 505, results in similar kinetics. Removing the quenching from the model has little effect on IsiA–PSI energy transfer (**Supplementary Table S2**), as has been experimentally observed (Chen et al., 2021).

The model calculations notably fail to reproduce the existence of multiple timescales of IsiA–PSI equilibration, differing by an order of magnitude, as resolved by the 2DES experiments. Therefore, we conclude that the multi-exponential or non-exponential kinetics are not intrinsic to individual PSI–IsiA complexes but reflects heterogeneity in the ensemble. There are several types of heterogeneity that could be regarded. Melkozernov et al. (2003) proposed that EET from IsiA to PSI occurs on two timescales – about 2 ps and 10 ps – they assign them to the existence of complexes with double IsiA rings around PSI, the shorter and longer lifetimes originating from the inner and outer ring respectively. On the other hand, structural fluctuations within individual complexes can also significantly affect the Chl—Chl interactions and disrupt or accelerate EET (Harris et al., 2023).

## Conclusions

In conclusion, the experimental 2DES results presented here show a remarkably fast EET from IsiA to the PSI core complex that, at least in a large fraction of the complexes, substantially exceeds the predictions from Förster energy transfer calculation. Our simplified Förster EET calculations predict that the rapid IsiA-PSI equilibration time is supported by multiple energy transfer routes between different IsiA subunits in the ring. A more accurate structure-based theoretical treatment taking into account the local environment effects on the electronic and electronic-vibrational interactions as well as fluctuations in the pigment and protein conformation might help understand the basis of the rapid EET and its lifetime heterogeneity. Evidently it would be helpful to acquire experimental data with higher signal-to-noise ratio and free from singlet annihilation – which might become attainable in the near future as the 2DES technology improves.

## Data availability statement

The raw data supporting the conclusions of this article will be made available by the authors, without undue reservation.

## Author contributions

PA: Conceptualization, Formal analysis, Funding acquisition, Investigation, Resources, Visualization, Writing – original draft. SJ: Investigation, Methodology, Writing – original draft. HT: Funding acquisition, Methodology, Supervision, Writing – review & editing. PL: Conceptualization, Formal analysis, Funding acquisition, Investigation, Software, Supervision, Writing – original draft.
