# Supplementary material for: Inhomogeneous energy transfer dynamics from iron-stress-induced protein A to photosystem I
Source: Front Plant Sci. 2024 May 16;15:1393886. doi: 10.3389/fpls.2024.1393886 (PMC11137255; doi:10.3389/fpls.2024.1393886)
Supplement: Supplementary file 1 [file DataSheet_1.pdf]

Supplementary Material

1 Supplementary Figures

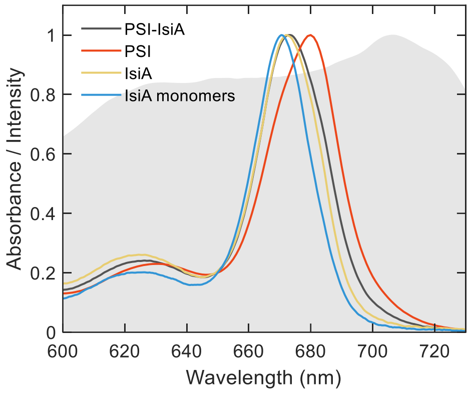

Supplementary Figure 1. Absorption spectra of isolated PSI–IsiA, trimeric PSI, IsiA aggregates and monomers and spectral profile of the excitation pulses used for 2DES.

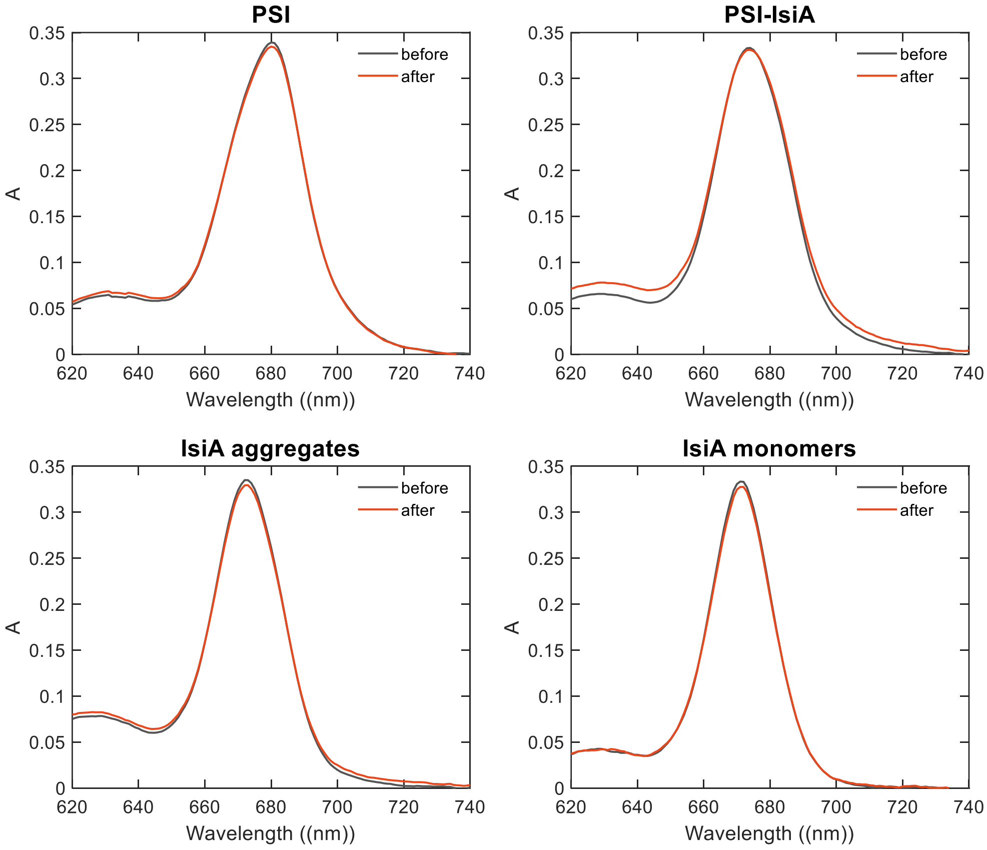

Supplementary Figure 2. Comparison of absorption spectra recorded from the samples before and after the 2D measurements.

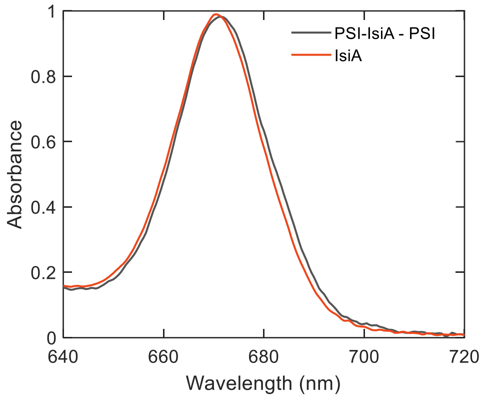

Supplementary Figure 3. Difference absorption spectrum of the PSI–IsiA supercomplex minus the trimeric PSI compared to the absorption spectrum of IsiA monomers. The spectra of PSI–IsiA, PSI and IsiA are area-normalized and then scaled by the estimated number of Chls in each (285 Chl *a* in a trimeric PSI and 306 in 18 IsiA).

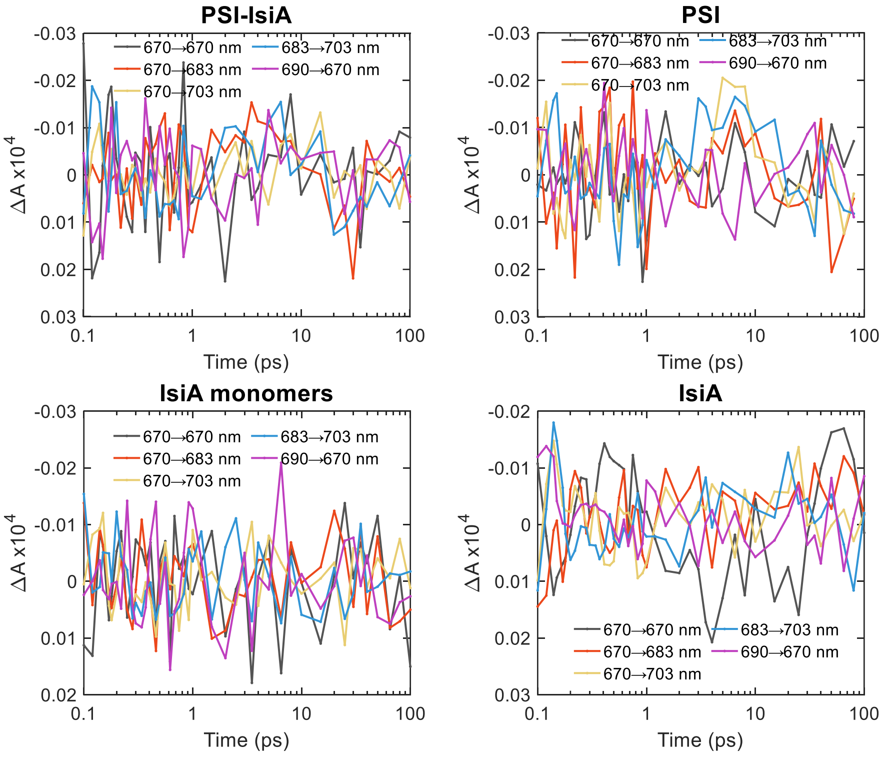

Supplementary Figure 4. Selected fit residuals for different excitation/detection wavelength pairs, obtained from global analysis of the 2DES data of PSI-IsiA, PSI, IsiA monomers and aggregates.

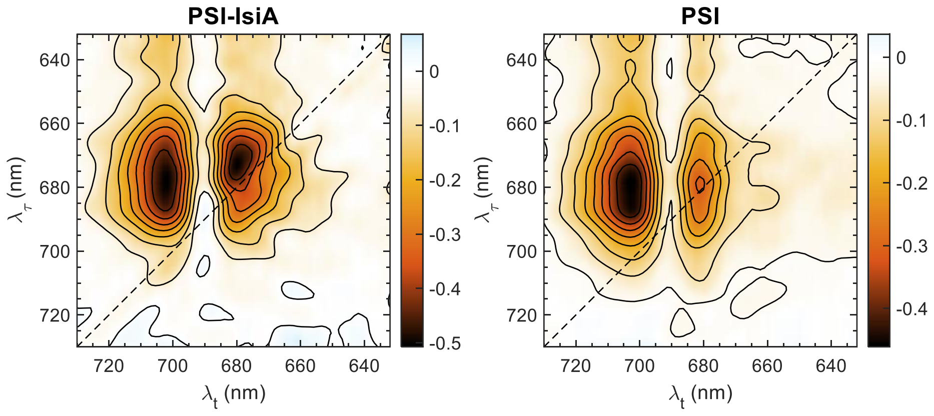

Supplementary Figure 5. 2D spectra of the non-decaying component of PSI-IsiA and PSI obtained by global analysis of the 2DES data.

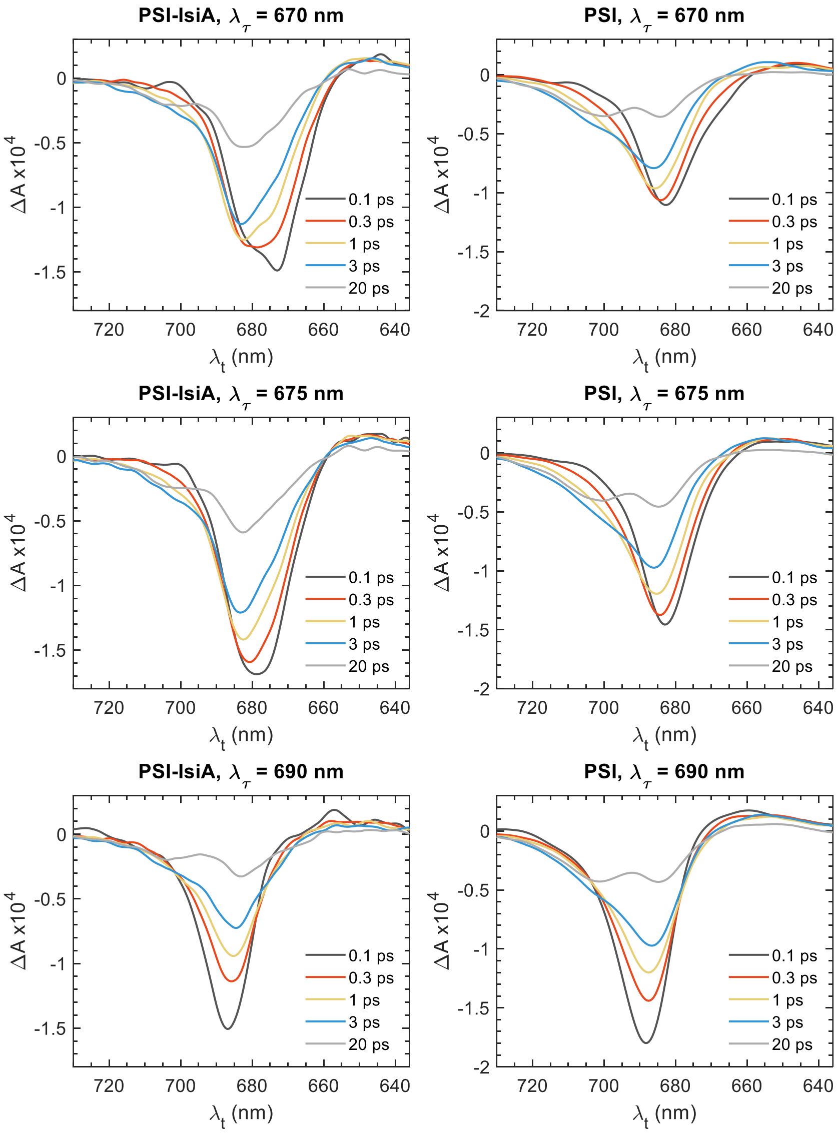

Supplementary Figure 6. Horizontal slices of the 2D electronic spectra of PSI–IsiA and PSI at different excitation wavelengths λ_τ.

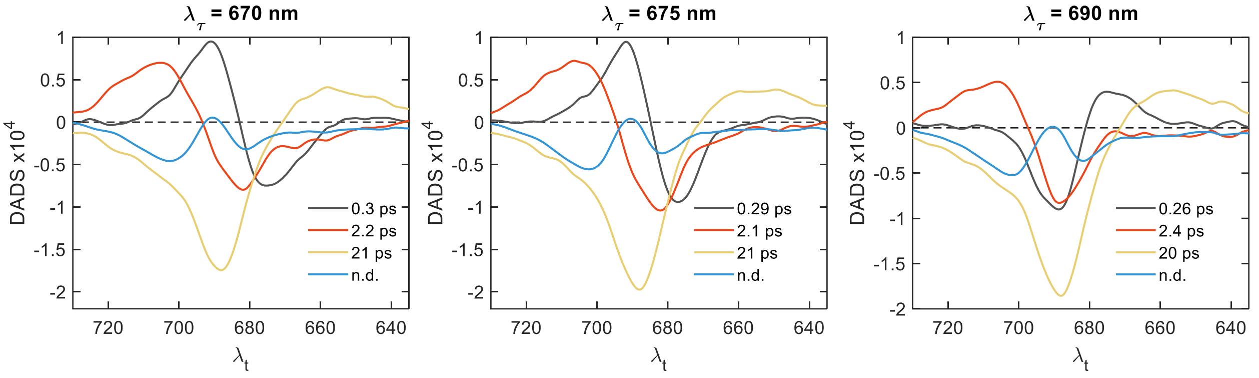

Supplementary Figure 7. Decay-associated spectra obtained by global analysis of horizontal slices of the 2D electronic spectra of PSI at different excitation wavelengths λ_τ.

2 Supplementary Tables

Supplementary Table 1. Fastest IsiA—IsiA and IsiA—PSI EET routes.

**IsiA—IsiA**		
a	***b***	k_{ab} **(ps^{-1})**
**W03**	y15	0.64
Z15	g03	0.55
X17	Y14	0.53
W15	X03	0.46
W17	X14	0.45
Z17	g14	0.37
Y17	Z14	0.26
X15	Y03	0.26
**IsiA—PSI**		
a	***b***	k_{ab} **(ps^{-1})**
g17	K03	0.23
Y14	J03	0.18
g08	K03	0.14
X14	F02	0.14
W14	B32	0.10
Z17	A14	0.08

Supplementary Table 2. Modelled IsiA and PSI population decay lifetimes.

**Model parameters**	τ_1 **(ps)**	τ_2 **(ps)**
Base PSI-IsiA model	12.1	36.6
Disabled IsiA excitation quenching	12.8	47.1
IsiA—IsiA EET 10-fold faster	11.9	36.2
Disabled fastest IsiA—PSI EET routes (cf. Table 1)	13.1	37.3
Disabled EET between IsiA g and PSI	15.6	38.0
Disabled EET between IsiA Y and PSI	14.4	38.0
Disabled EET between IsiA g,Y,W and PSI	19.0	43.5

3 Supplementary Methods

3.1 Absorption spectroscopy

Absorption spectra in the range of 350–750 nm were recorded at room temperature with a dual-beam spectrophotometer (Ocean Insight). The measurements were performed in a quartz cuvette of 1-mm optical path length with 1 nm spectral bandwidth.

3.2 Förster energy transfer calculation

The PSI energy transfer dynamics was modelled using simplified Förster energy transfer calculations as outlined by Harris et al. (2023). To obtain a more realistic dynamics, we include minimal modifications to the model:

1) A decay rate constant of 0.5 ns^{-1} was added to all Chls in PSI. A decay rate constant of 0.1 ps^{-1} was added to the IsiA Chls to simulate non-photochemical quenching in IsiA.

2) The ratio of the inward/outward EET rate constants between IsiA and PSI Chls was set to 3 to account for the overall difference in excitation energy. The ratio is based on estimation of the first spectral moments of the fluorescence emission spectra of IsiA and PSI being around 692 and 701 nm, respectively, which corresponds to an energy difference of ~20 meV. Consequently, the forward/reverse ratio satisfying the Bolzmann equilibrium at room temperature is between 2 and 3. The overall population of IsiA in the transfer equilibrium state is between 40% and 50%, which is in the range of values obtained by fitting the fluorescence emission spectrum of PSI-IsiA.

3) An irreversible trapping rate constant of 10 ps^{-1} was added to the reaction centre Chls. This value probably overestimates the actual microscopic rate constant of charge separation, however, it is phenomenologically adjusted such that the effective trapping lifetime of the PSI core complex is approximately equal to the measured trapping lifetime (25 ps).

To estimate the population kinetics, the rate equation of the entire system is solved by eigendecomposition of the matrix of rate constants **K,** where element k_{ij} is the rate constant from the jth to the ith pigment and $-k_{ii}$ is equal to the sum of all rate constants out of the ith pigment.
